# O.2.2-11 Adapting national physical activity guidelines based on the global WHO guidelines: experiences and challenges from the case of Switzerland

**DOI:** 10.1093/eurpub/ckad133.126

**Published:** 2023-09-11

**Authors:** Sonja Kahlmeier, Anja Frei, Susi Kriemler, Claudio Nigg, Thomas Radtke, Katja Manike, Simon Endes

**Affiliations:** Department of Health, Swiss Distance University of Applied Science FFHS, Switzerland; Institute for Epidemiology, Biostatistics and Prevention, University of Zurich; Institute for Epidemiology, Biostatistics and Prevention, University of Zurich; Institute of Sport Science, University of Berne, Switzerland; Institute for Epidemiology, Biostatistics and Prevention, University of Zurich; Department of Health, Ecoplan, Switzerland; sonja.kahlmeier@ffhs.ch; Department of Health, Ecoplan, Switzerland; sonja.kahlmeier@ffhs.ch

## Abstract

Physical activity guidelines are recognized as an important element of a national approach to promote physical activity. This publication summarizes the approach and process taken to update the Swiss Guidelines, discusses experiences and identifies challenges. The multistage project involved to: 1) summarize the scientific evidence underpinning the 2020-edition of the WHO guidelines; 2) to systematically analyze the existing Swiss guidelines for different target groups and to develop proposals for updates 3) a participatory process to gain consensus with the main interested groups 4) to finalize the guidelines. Updated guidelines were adopted for infants, pre-school children, children and young people, adults, older adults and pregnant and postpartum women, in most cases adopting the WHO guidelines. Children, young people and adults living with disability and adults and older adults with chronic conditions are specifically addressed in each of the general guidelines, rather than adopting separate guidelines for each of these groups as done by the WHO. The systematic approach in identifying aspects to update, the participatory approach and a strong scientific consortium with different thematic backgrounds were key strengths in the process. Challenges included the large amount of feedback and finding scientifically sound compromises. The updated versions of the Swiss national guidelines for small children, young people, adults and women during and after pregnancy provide an excellent basis to further promote physical activity in Switzerland. A remaining key task is to develop a range of communication tools and materials for different target groups beyond the circle of experts and interested groups, considering available evidence on optimal messaging and best outlet tools and channels.

